# Chicken skin-derived collagen peptides chelated zinc promotes zinc absorption and represses tumor growth and invasion *in vivo* by suppressing autophagy

**DOI:** 10.3389/fnut.2022.960926

**Published:** 2022-08-03

**Authors:** Tengfei Liu, Lifang Zou, Xiaowen Ji, Guiran Xiao

**Affiliations:** ^1^China Light Industry Key Laboratory of Meat Microbial Control and Utilization, Hefei University of Technology, Hefei, China; ^2^School of Food and Biological Engineering, Hefei University of Technology, Hefei, China

**Keywords:** collagen peptides, chicken skin, zinc, tumor, autophagy

## Abstract

To improve the utilization value of chicken by-products, we utilized the method of step-by-step hydrolysis with bromelain and flavourzyme to prepare low molecular weight chicken skin collagen peptides (CCP) (<5 kDa) and characterized the amino acids composition of the CCP. Then, we prepared novel CCP-chelated zinc (CCP–Zn) by chelating the CCP with ZnSO_4_. We found that the bioavailability of CCP–Zn is higher than ZnSO_4_. Besides, CCP, ZnSO_4_, or CCP–Zn effectively repressed the tumor growth, invasion, and migration in a *Drosophila* malignant tumor model. Moreover, the anti-tumor activity of CCP–Zn is higher than CCP or ZnSO_4_. Furthermore, the functional mechanism studies indicated that CCP, ZnSO_4_, or CCP–Zn inhibits tumor progression by reducing the autonomous and non-autonomous autophagy in tumor cells and the microenvironment. Therefore, this research provides *in vivo* evidence for utilizing chicken skin in the development of zinc supplements and cancer treatment in the future.

## Introduction

Zinc is the second most abundant micronutrient in humans and plays critical roles in many general cellular functions (1). The distribution, regulation, and crosstalk of these micronutrients are widely involved in various physiological and pathological processes (2–4). The significance of zinc for health has been paid more and more attention because its deficiency may lead to many diseases, such as anorexia, loss of appetite, smell and taste disorders, and other disorders (4–6).

The relationship between zinc and tumor has been intensively investigated in experimental and human studies over decades (4, 7, 8). For example, many studies demonstrated reduced zinc levels in different cancers and the corresponding downregulation of zinc importers and the upregulation of zinc exporters (7, 9). Large numbers of epidemiological and clinical studies demonstrated that zinc deficiency is associated with an increased risk of some cancers, such as prostate, lung, esophageal, and oral cancers (10–12). Moreover, the zinc supplement therapeutic method has been proven to be good tumor adjuvant therapy. For example, zinc-L-carnosine, which refers to one chelation-based compound containing L-carnosine as well as zinc, showed a well-alleviating effect on the side-effects of cancer treatment (4, 13). ZnO nano-scale particles were able to target multiple cell types of carcinoma, covering carcinoma cells, carcinoma stem cells, and macrophages, and simultaneously perform a number of vital roles, including limitation of carcinoma proliferating process, sensitization of drug-resistant carcinoma, prevention of carcinoma recurrence and metastasis, and resuscitation of carcinoma immunosurveillance (14). These data strongly suggested that zinc may play a protective role in the formation and progression of different cancer processes; however, the underlying mechanisms have not been fully understood.

Zinc deficiency would emerge quickly along with an inappropriate diet (15). The main causes of zinc deficiency are as follows: insufficient intake, decreased absorption, increased loss, or increased demand (16). Zinc deficiency could be supplemented by zinc-rich foods or zinc supplements. Zinc generally combines with small peptides through coordination bonds and ionic bonds to form a unique stable structure of a five-membered ring or six-membered ring in which ionic bonds and coordination bonds coexist (17). The new-generation zinc supplements, which refer to a chelation-based compound containing polypeptide or an amino acid as well as zinc, have attracted more and more attention because of the advantages of high bioavailability and low toxicity.

Collagen is a fibrous structural protein with two α (I) chains and one α (II) chain intertwined in a certain way to form a triple helix structure (18). Collagen has a large molecular weight and is difficult to be directly absorbed and utilized, whereas the small molecular collagen peptides after collagen hydrolysis have higher absorption efficiency and stronger biological activity (19). Therefore, collagen peptides have been widely applied in food, medicine, tissue engineering, cosmetics, and other fields (20). In the past decades, collagen hydrolysates from bovine bone gelatin have attracted great scientific and industrial interests with potential use in various health-related sectors. In recent years, religious beliefs and disease concerns (such as mad cow disease) have gradually changed people’s research interests toward the development of collagen and its products from terrestrial mammals to poultry and marine organisms (21). Using protease to hydrolyze by-products and produce collagen peptides not only reduces wastage of resources and environmental pollution but also allows the efficient utilization of by-products and diversifies the types of collagen peptides (22, 23). The consumption of chicken is growing rapidly in the world. As the main by-product of poultry processing, chicken skin is roughly processed to produce feed or low-grade minced meat at present. Chicken skin is rich in collagen and is a promising source of collagen products (24). Collagen peptides extracted from chicken skin and chelated with zinc cannot only improve the utilization of chicken skin but also provide a new reference for the research of new-generation zinc supplements.

*Drosophila melanogaster* (hereinafter *Drosophila*) is one of the most studied eukaryotic organisms and has made fundamental contributions to different areas of biology and human diseases studies (25). Up to 75% of the human genes implicated in diseases are conserved in *Drosophila* (26). In this study, we used chicken skin as raw materials, extracted small-molecular-weight chicken collagen peptides (CCP) with high zinc-chelating ability, and performed chelation of CCP with ZnSO_4_ to prepare novel CCP-chelated zinc (CCP–Zn). Using a zinc-deficient *Drosophila* model [zinc depletion in food through the addition of 50 μM zinc-specific chelator N,N,N’,N’-tetrakis (2-pyridylmethyl) ethylenediamine (TPEN) (27)], we evaluated the bioavailability of the prepared CCP–Zn. Next, using a *Drosophila* model of malignant tumor, wherein the activated oncogene Raf (Raf^GOF^) cooperates with loss-of-function mutations in the conserved tumor suppressor scribble (scrib^–/–^) (28), we evaluated the effect of oral administration of the CCP–Zn on tumor progression in *Drosophila* and determined the relevant mechanism.

## Materials and methods

### *Drosophila* strains and culture media

Stocks were normally reared on standard cornmeal media or drug-contained media at 25^°^C and 60% humidity on a 12 h light:12 h dark cycle. All fly crosses and larvae were maintained in vials containing traditional corn-yeast media (normal food, NF) or drug-contained media. The corn-yeast standard food was prepared according to the traditional corn-yeast media: 100 g corn, 10 g soybean meal, 40 g brown sugar, 14.5 g sugar, 25 g yeast, 8.0 g agar mixed with hot water to make a 1,000 ml diet. Crosses were flipped directly onto the drug-containing medium. The concentrations of supplemented chicken skin collagen peptides (CCP), zinc sulfate (ZnSO_4_, Cat#221376, Sigma-Aldrich Corporation), or chicken skin collagen peptides–zinc (CCP–Zn) used were as follows: 1.28 g/L CCP, 3 mM ZnSO_4_, 1.28 g/L CCP–Zn (contains 3 mM ZnSO_4_). *w*^1118^ (V#60000) was obtained from the Vienna *Drosophila* RNAi Center. Atg8a-mCherry, y,w,ey-flp; Act-y^+^-Gal4 UAS-GFP; FRT82B tub-Gal80 and w; Adv/Cyo; UAS-Raf^GOF^ FRT82B scrib1/TM6B were generously provided by Dr. Jose C. PASTOR-PAREJA (29, 30).

### Enzymatic hydrolysis of chicken skin collagen

After removing fat and impurities from the chicken skin, ultrapure water was added to the chicken skin, and the ratio of material to liquid was 1:10 (g/ml). Flavourzyme and bromelain were used to hydrolyze the chicken skin collagen step by step. To improve the efficiency of the enzymatic hydrolysis reaction, the effects of hydrolysis pH, temperature, reaction time, substrate concentration, and the enzyme amount on the degree of hydrolysis (DH) and extraction rate of peptides were optimized in this study. Overall, the optimum conditions for the enzymatic hydrolysis of chicken skin collagen were obtained at the hydrolysis temperature of flavourzyme which is 60°C, at pH 7.0, and the additional amount and the hydrolysis time of flavourzyme is 3% and 4 h. After that, the hydrolysate was heated at 100°C for 10 min, then 3% bromelain was added, the hydrolysis temperature was set at 55^°^C, pH 6.5, and the hydrolysis time of bromelain is 4 h.

### Peptide separation by size-exclusion chromatography

The CCP were prepared with a concentration of 0.1 g/ml. Then, the samples were subjected to size exclusion chromatography (SEC) using a Sephadex column (10 × 300 mm) packed with Cytiva Superdex™ 200 increase 10/300 GL (Cytiva, United States) to fractionate the peptides according to their molecular masses. The separation was performed at a constant flow rate of 0.4 ml min^–1^ with 0.01 M HCl at room temperature. The fractions were assayed using an ultraviolet detector (Amersham Biosciences) at 214 nm. Standards with different MW were used, such as Insulin (5,807.6215 Da), bacitracin (1,423 Da), glycyl-proline-glycyl-alanine-glutamine-glycyl-arginine-proline-proline (835.912 Da), and glycyl-glycyl-leucine (245.278 Da) for the analytical curve (log MW × volume), and the calculation of the MW distribution was performed by using volume range percentage.

### Degree of hydrolysis assay

The DH was calculated using the relationship between amino nitrogen (AN) and total nitrogen (TPN). AN was tested by the formaldehyde titration method, and TPN was tested by the *Kjeldahl* method (31). DH value was calculated using the following Equation:


$$\mathrm{DH}\left(\%\right)=\frac{\text{AN}}{\text{TPN}}\times 100\%$$

### The extraction rate of peptides

The content of peptides was determined by the folin phenol reagent as previously described (32). Briefly, trichloroacetic acid (TCA) was added to a final concentration of 10% (w/w). The mixture was vortexed, stored for 10 min, centrifuged for 10 min at 10,000 *g*, and the supernatant was collected. Bovine serum albumin was used as the standard to draw the standard curve.

### Preparation of chicken skin collagen peptides–zinc

To prepare the CCP–Zn complexes, the mass ratio of CCP and ZnSO_4_ was 0.88:1, and the mixture was incubated in a 43.6°C water bath for 42.5 min after the pH of the reaction was adjusted to 6.57. The mixture was washed with ethanol to remove free zinc ions, then lyophilized, and stored at −20°C for further use.

### Determination of amino acid composition

The CCP and CCP–Zn (0.1 g) were hydrolyzed with a 6 M hydrochloric acid solution in the sand bath at 110°C for 16 h, and then a citrate buffer solution was added. After filtration through a 0.22 μm microporous membrane, the amino acid composition of CCP and CCP–Zn was analyzed by an automatic amino acid analyzer (Sykam S-433D, Germany).

The hydroxyproline (Hyp) content of CCP and CCP–Zn was estimated according to GB/T 9695.23–2008, National Standards of People’s Republic of China. Briefly, CCP and CCP–Zn (0.1 g) were hydrolyzed with a 6 M hydrochloric acid solution in the air oven at 110°C for 16 h; the samples were placed in a 50 ml volumetric flask. Then, 1 ml of the sample was mixed with 1 ml of chloramine T solution (50 mM) and was allowed to stand for 20 min at 25°C. Subsequently, 2 ml of p-dimethylaminobenzaldehyde–perchloric acid–isopropyl alcohol solution (0.67 M, 65 ml isopropyl alcohol was added in 35 ml perchloric acid solution) was added to each sample and heated at 60°C for 20 min in a water bath. The samples were cooled in running water for 2 min, stored for 30 min at 25°C, and the hydroxyproline content was determined based on the absorbance (at 558 nm) obtained using a hydroxyproline standard curve.

### Ultraviolet spectrometry

Ultraviolet spectrometry was performed as described previously (33). The UV spectra of CCP and CCP–Zn were recorded at room temperature on TECAN Infinite M nano equipment (Switzerland). The scan range was 190–330 nm.

### Fourier transform infrared spectrometry

Fourier transform infrared spectrometry was performed as described previously (34). In total, 1 mg of freeze-dried CCP and CCP–Zn were mixed with 100 mg of dry KBr. The mixture was ground into a fine powder and compressed into a thin disc. All FTIR spectra were recorded by an FT-IR spectrometer (Thermo Nicolet, United States) over a wavenumber region between 4,000 and 600 cm^–1^ at a resolution of 10 cm^–1^.

### Particle size measurement

Particle size measurement was performed as described previously (35). The CCP or the CCP–Zn was dissolved in deionized water and equilibration for 5 min at room temperature. The particle size was measured by MS 2000 (Malvern Instruments Company of United Kingdom) at room temperature.

### Zinpyr-1 staining assay

Zinpyr-1 staining assay was performed as described previously (36). For Zinpyr-1 staining, the fat bodies of third instar larvae were dissected and fixed in 4% formaldehyde in PBS for 10 min at room temperature, 2 μM Zinpyr-1 stained for 60 min at room temperature, washed thrice with PBS, and mounted with 50% glycerol/PBS. The fluorescence signal was examined by a fluorescence microscope (Nikon, Ti2, Japan).

### Oxidation radical scavenging rate

(1) DPPH (2,2-Diphenyl-1-picrylhydrazyl) scavenging: Chicken skin collagen polypeptide freeze-dried powder was taken, collagen peptide solutions with different concentrations were prepared, then 2 ml DPPH solution and 2 ml sample solution were taken, shaken well, reacted in darkness for 30 min, and finally their absorbance were measured at 517 nm wavelength (37, 38). The inhibition rate is calculated as follows:


$$\mathrm{DPPH}\mathrm{clearance}\left(\%\right)=\frac{\mathrm{A0}-\left(\mathrm{Ai}-\mathrm{Aj}\right)}{\mathrm{A0}}\times 100\%$$

Ai is the mixed solution of DPPH and sample; Aj is the mixed solution of ethanol and sample; A0 is a mixed solution of DPPH and water.

(2) OH-scavenging: In total, 16 ml of salicylic acid ethanol solution with a concentration of 6 mM, 2 ml of ferrous sulfate solution with a concentration of 6 mM, and 2 ml of H_2_O_2_ aqueous solution with a concentration of 6 mM were mixed with 5 ml of polypeptide solutions with different concentrations. The mixture was placed in a water bath at 37°C for 15 min, and the absorbance was measured at 510 nm (A_*X*_).

Using 5 ml distilled water instead of polypeptide solution, the absorbance value was determined according to the above method and recorded as a blank (A_0_). The absorbance was determined using 2 ml of distilled water instead of 2 ml of 6 mM H_2_O_2_ aqueous solution, according to the above method and recorded as a control (A_*X0*_). The formula for calculating the scavenging rate of hydroxyl radical (OH^–^) is as follows:


$${\text{OH}}^{-}\mathrm{clearance}\left(\%\right)=\frac{{\text{A}}_{0}-\left({\text{A}}_{\text{X}}-{\text{A}}_{\mathrm{X0}}\right)}{{\text{A}}_{0}}\times 100\%$$

### Statistics of body indexes of *Drosophila* larvae

#### *Drosophila* larvae length

A total of 20 third-instar *Drosophila* larvae were selected and cleaned with PBS. The *Drosophila* larvae were put in a 4^°^C refrigerator for 30 min to weaken their mobility. They were photographed with a camera, and then the length of *Drosophila* larvae was counted by the ImageJ software.

#### *Drosophila* larvae weight

A total of 30 third-instar *Drosophila* larvae were selected and cleaned with PBS, the water on the larvae surface was sucked dry, and then their weights were weighed.

### Ribonucleic acid isolation and semiquantitative RT–PCR

Unless otherwise noted, ribonucleic acid (RNA) was made from 15 third-instar larvae. Semi-quantitative RT–PCR was performed using the specific primers corresponding to partial regions of target genes. Unless otherwise noted, RNA was made from 15 third-instar larvae. Semi-quantitative RT–PCR was performed using the specific primers corresponding to partial regions of target genes. The ribosomal protein 49 gene (rp49) was used as the control. RNA isolation and reverse transcription were performed independently three times, and no less than three PCR experiments were applied to each cDNA sample. The primers used for RT–PCR were as follows:

rp49-F: 5′-GCACCAAGCACTTCATCC-3′

rp49-R: 5′-CGATCTCGCCGCAGTAAA-3′

MtnB-F: 5′-TCGCCTCAGCCAAGTGAAAG-3′

MtnB-R: 5′-CCCATTCTTGCAAACGCACT-3′.

### Detection of zinc supplement effect

Wild-type *Drosophila* melanogaster *W*^1118^ was fed on *Drosophila* maize medium supplemented with 50 μM TPEN. After its first generation, *Drosophila melanogaster* grew to the third-instar larva stage, it was picked out and transferred to the medium supplemented with CCP (1.28 g/L), ZnSO_4_ (3 mM), or CCP–Zn (1.28 g/L). This ensures that they contain the same amount of zinc. After growing to the wandering stage, the zinc level in its body was detected.

### Whole-body fluorescence imaging of *Drosophila*

Twelve days after egg laying (AEL) *Drosophila* larvae were selected, 1 × PBS was used to clean the food attached to the body surface of *Drosophila* larvae, and then the washed larvae were placed on a slide. To prevent the larvae from twisting and crawling during the shooting process, a slide cover was taken and then pressed. The process should be gentle to prevent damage to *Drosophila* body tissues or organs, and only one larva should be placed on each slide.

### Statistical analysis

Tumor size and fluorescence intensity were measured with the ImageJ software. Quantification of the data was presented in bar graphs created with GraphPad Prism 5. Data were analyzed by Student’s *t*-test between groups, and one-way analysis of variance (ANOVA) was used for multiple groups. Statistical results were presented as means ± SEM. Asterisks indicate critical levels of significance (*P < 0.05, ^**^P < 0.01, and ^***^P < 0.001).

## Results

### Collagen peptides from chicken skin using commercial enzymes showed antioxidative activity

**FIGURE 1:**
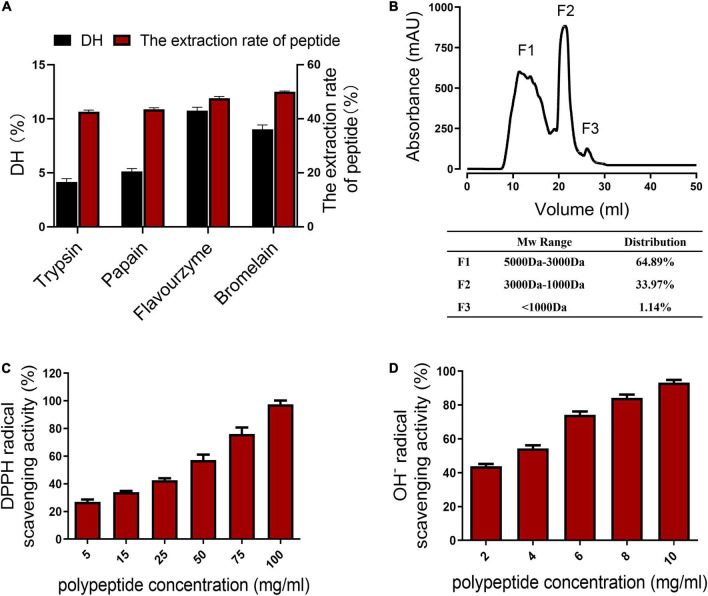
Properties of the chicken skin collagen peptides (CCP). **(A)** Degree of hydrolysis (DH) of the collagen hydrolysates produced by trypsin, papain, flavourzyme, and bromelain. **(B)** The molecular weight profiles of the chicken skin collagen hydrolysates were determined by the size-exclusion chromatography. The molecular weights of the CCP are smaller than 5 kDa. **(C)** DPPH radical scavenging activity of the CCP. The clearance of CCP to DPPH free radicals is concentration dependent. **(D)** The clearance of CCP to OH^–^ is concentration dependent.

The chicken skin collagen was hydrolyzed with four commercial proteases, including trypsin, papain, flavourzyme, and bromelain. Finally, bromelain and flavourzyme were used to hydrolyze chicken skin collagen step by step because they showed the highest degree of hydrolysis (DH) and extraction rate of peptides (Figure 1A). After treating chicken skin collagen with the double enzymes step hydrolysis, the molecular weight profiles of the chicken skin collagen hydrolysates were determined by size-exclusion chromatography (Figure 1B). The molecular weight distribution of CCP is shown in Figure 1B. The CCP was mainly composed of peptides with a molecular weight of 3,000–5,000 Da, which accounted for 64.89%. The percentage of peptides with a molecular weight of 1,000–3,000 Da in the CCP is about 33.97% (Figure 1B).

Besides, the CCP exhibited scavenging activities against DPPH free radicals and hydroxyl-free radicals (OH^–^). As shown in Figure 1C, the concentration of the CCP is positively correlated with the scavenging efficiency of DPPH. The results showed that DPPH clearance rate and peptide concentration (5–100 mg/ml) conform to the linear Equation *y* = 0.0073 *x* + 0.2292 (*R*^2^ = 0.9968). The ability of the peptides to scavenge OH^–^ is shown in Figure 1D. The concentration of the CCP is also positively correlated with the OH^–^ scavenging rate. The OH^–^ scavenging rate and the peptides concentration (2–10 mg/ml) conform to the linear Equation *y* = 0.0649 *x* + 0.3013 (*R*^2^ = 0.9903). These results showed that the inhibition of these peptides on the DPPH free radicals and OH^–^ with higher concentrations of samples increased. Moreover, the CCP exhibited a strong scavenging effect on OH^–^, but the DPPH radical scavenging activity of the CCP is low.

### The preparation of chicken skin collagen peptides–zinc by chelating the chicken skin collagen peptides with zinc sulfate and the amino acid composition of chicken skin collagen peptides and chicken skin collagen peptides-zinc

**FIGURE 2:**
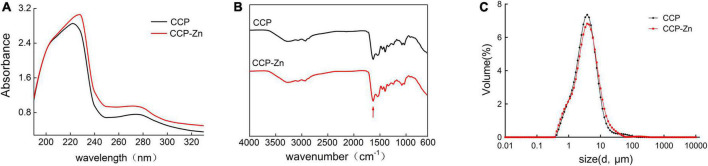
Characterization of the CCP and CCP-chelated zinc (CCP–Zn). **(A)** The CCP and synthesized CCP–Zn are characterized using ultraviolet–visible spectroscopy. **(B)** The CCP and CCP–Zn are characterized by Fourier Infrared (FTIR). **(C)** The CCP and CCP–Zn showed different particle sizes.

Subsequently, novel CCP–Zn was prepared by chelating the CCP with ZnSO_4_, zinc content of the CCP–Zn was 152.8 mg/g. Characterizations of the CCP and CCP–Zn are shown in Figure 2. After chelating with ZnSO_4_, the maximum absorption wavelength was shifted because the structure of CCP–Zn changed, and valence electron transition occurred (Figure 2A). The ultraviolet–visible spectroscopy results that the maximum absorption wavelength of CCP is about 220 nm, but CCP–Zn is shifted to 230 nm (Figure 2A). The structures of the CCP and CCP–Zn were characterized by Fourier infrared (FTIR) (Figure 2B). Untreated infrared spectrograms of CCP and CCP–Zn are shown in Figure 2B. The data showed that the shape of the spectrum changes a little before and after the chelation reaction of CCP with zinc, but after the chelation reaction, the infrared absorption of the sample changes around 1,650 cm^–1^ (Figure 2B). The characteristic absorption of C = O stretching vibration of the amide I band is around 1,650 cm^–1^ (37). This suggests that the carboxyl group on CCP combines with zinc. Besides, CCP and CCP–Zn showed different particle sizes (Figure 2C). The particle size distribution of CCP is 0.363–138.03 μm, mainly distributed in 2.18–7.58 μm; the overall particle size of CCP–Zn is larger than that of CCP (Figure 2C). All these data suggested that CCP–Zn was made successfully.

According to previous studies, the zinc-binding activity of peptides is closely related to their molecular weight, amino acid sequence composition, and specific groups and residues (38). Many studies have shown that hydrophilic amino acids, including aspartic acid, arginine, histidine, serine, glutamic acid, lysine, and cysteine are correlated with metal-binding activities (39, 40). The amino acid composition of CCP and CCP–Zn was analyzed and listed in Table 1. In terms of CCP and CCP–Zn, the glycine content > 20% was the highest, the content of proline, hydroxyproline, and alanine were about 10%, and the results are consistent with collagen peptides. The total contents of glutamic acid, aspartic acid, histidine, lysine, and arginine in the CCP–Zn chelate significantly increased from 20.12 to 21.76% after chelating with zinc. The finding suggested that acidic amino acids and basic amino acids participated in the formation of the CCP–Zn chelate. These data indicated that chicken skin-originated collagen peptides showed good potential as zinc carriers for further investigation.

**TABLE 1:** The amino acid composition of the chicken skin collagen peptides (CCP) and CCP-chelated zinc (CCP–Zn).

Amino acids	CCP (%)	CCP–Zn (%)
Aspartic acid	2.84	3.72
Threonine	3.86	4.70
Serine	3.89	4.70
Glutamic acid	7.98	8.04
Glycine	23.00	21.99
Alanine	10.19	9.52
Cysteine	0.43	0.55
Valine	5.36	3.57
Methionine	3.19	1.83
Isoleucine	1.14	1.46
Leucine	1.70	2.19
Tyrosine	3.14	3.47
Phenylalanine	2.66	2.11
Lysine	2.53	2.83
Histidine	3.11	3.04
Arginine	3.65	4.12
Proline	10.48	11.48
Hydroxyproline	10.83	10.69

### The zinc-chelating chicken skin collagen peptides have higher bioavailability than inorganic zinc

**FIGURE 3:**
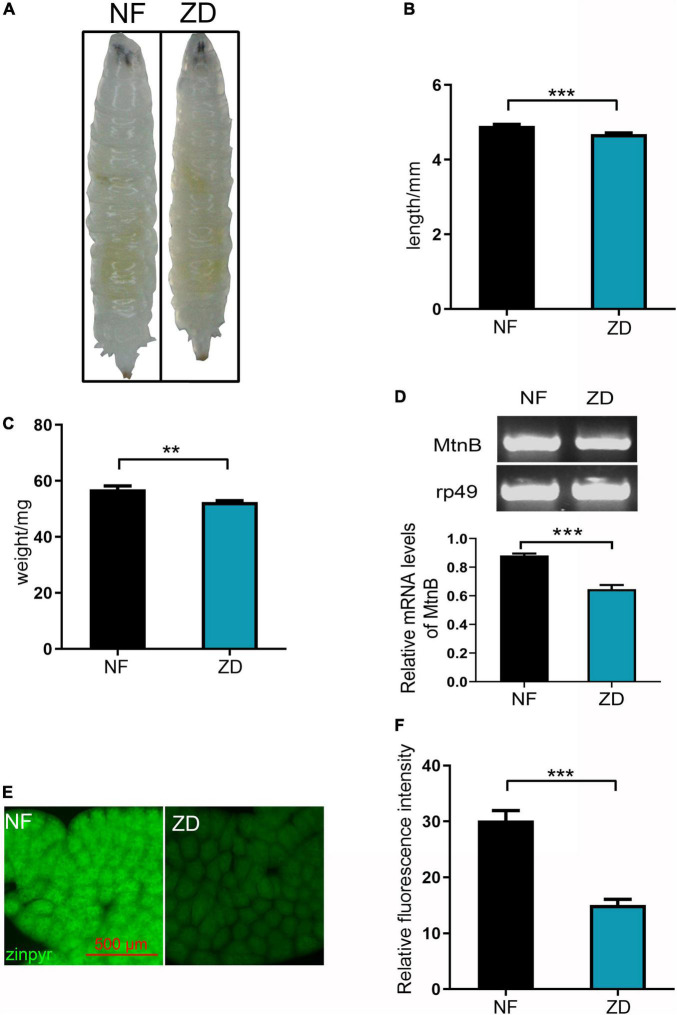
Establishing an experimental model of zinc deficiency in *Drosophila.* **(A)** Compared with the control group, the zinc-deficient third instar larva (*Drosophila* zinc deficiency model) is thinner. **(B)** The body length of the *Drosophila* zinc deficiency model is shorter than that of the control group, *n* = 30. **(C)** The weight of the *Drosophila* zinc deficiency model is lighter than that of the control group, *n* = 30. **(D)** The expression level of Mtnb in the *Drosophila* zinc deficiency model was decreased, *n* = 10. **(E)** Zinpyr-1 staining showed that zinc levels in the fat body of the *Drosophila* zinc deficiency model was decreased, *n* = 10. **(F)** Quantitative measurement of the fluorescent signals in **(E)**. Data are represented as mean ± SEM of the biological replicates. **p < 0.01, ***p < 0.001; two-tailed Student’s *t*-test.

It has been reported that the stability, absorption, and utilization of organic zinc are better than inorganic zinc. To assay the bioavailability of the CCP–Zn *in vivo*, a zinc-deficient *Drosophila* model was established to test whether its zinc supplement efficiency is better than ZnSO_4_. Wild-type *Drosophila* was reared in food supplemented zinc chelator [50 μM N,N,N’,N’-tetrakis (2-pyridylmethyl) ethylenediamine (TPEN)], and the progeny were laid and reared on this zinc deficient food, so these progenies are zinc deficient *Drosophila* model. It is known that the inhibition of growth is a cardinal symptom of zinc deficiency (41). As shown in Figure 3A, the third-instar larvae fed on TPEN is thinner than the normal larvae. Besides, the size of the zinc deficient *Drosophila* model is smaller than control (Figure 3B), and the weight of the zinc deficient *Drosophila* model is lower (Figure 3C). Metallothionein B (MtnB) expression has been previously reported to be sensitive to intracellular zinc levels and is considered a sensitive indicator of intracellular zinc levels (42). To detect intracellular zinc levels, the mRNAs in the whole body of the third instar larvae were extracted to detect MtnB expression. As shown in Figure 3D, the MtnB mRNA level was significantly decreased in the zinc-deficient model compared with the control. Zinpyr-1 is a zinc sensor that is particularly amenable to intracellular work in response to intracellular zinc (43, 44). As shown in Figures 3E,F, the zinc signal in the cytoplasm of *Drosophila* larvae grown on zinc-deficient food is reduced by about 50% compared with *Drosophila* larvae fed normal food. These results suggest that zinc level in *Drosophila* larvae fed on 50 μM TPEN is lower. Taken together, the zinc deficient *Drosophila* model was established successfully.

**FIGURE 4:**
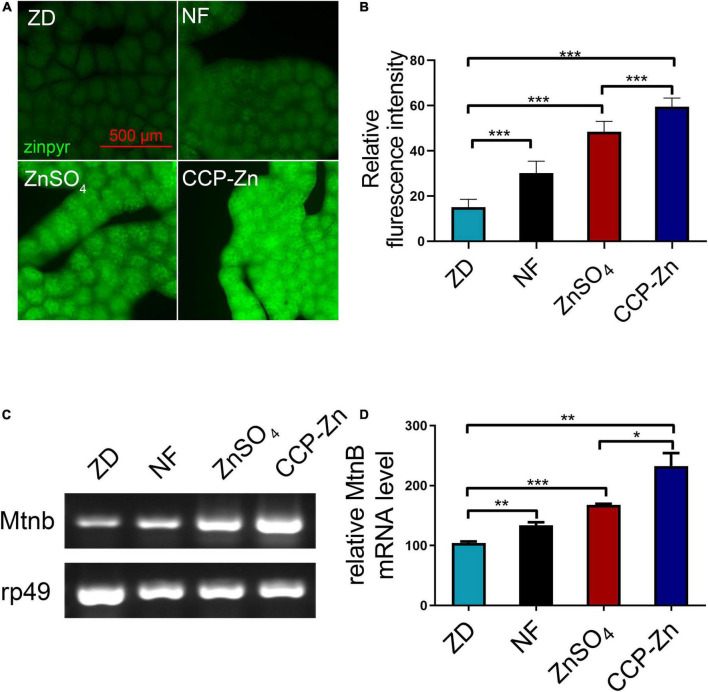
The bioavailability of CCP–Zn is higher than ZnSO_4_. **(A)** Zinpyr-1 staining showed that the zinc levels in the fat body of *Drosophila* raised on CCP–Zn is higher than flies raised on CCP–Zn (1.28 g/L) or ZnSO_4_ (3 mM), *n* = 10. **(B)** Quantitative measurement of the fluorescent signals in **(B)**. **(C)** RT-PCR analysis indicated that MtnB mRNA levels in the whole body of *Drosophila* raised on CCP–Zn (1.28 g/L) are higher than flies raised on ZnSO_4_ (3 mM), *n* = 10. **(B,D)** Quantitative measurement of **(C)**. Data are represented as mean ± SEM of the biological replicates. *p < 0.05, **p < 0.01, ***p < 0.001; two-tailed Student’s *t*-test.

To study if the bioavailability of the organic zinc produced is better, the third-instar zinc-deficient larvae fed on zinc-reduced foods were transferred to normal foods, food supplemented with 3 mM ZnSO_4_, or 1.28 g/L CCP–Zn (contains 3 mM ZnSO_4_). The zinc-deficient larvae grew well in the zinc supplementation food. The inhibition of growth phenotypes was disappeared, containing thinner, smaller size, and lower weight. Then, the zinc levels in the body were detected in these larvae (Figure 4). The zinc staining data showed that the zinc levels in fat body cells are greatly increased in larvae transferred to food supplemented with ZnSO_4_ or CCP–Zn (Figures 4A,B). Compared with the ZnSO_4_ supplementation group, the zinc levels in the fat body cells of larvae fed with CCP–Zn are increased by about 22.9% (Figure 4B). Besides, MtnB expression levels in the whole body are induced in larvae fed with ZnSO_4_ or CCP–Zn, and MtnB mRNA levels in CCP–Zn group exhibited 38.7% more than ZnSO_4_ group. These data suggested that CCP–Zn is easier to be absorbed than ZnSO_4_. Taken together, these results indicated that CPP-Zn has higher bioavailability and better zinc supplement efficiency than inorganic zinc (ZnSO_4_).

### The inhibitory effect of chicken skin collagen peptides–zinc on tumor growth, invasion, and distant migration is better than chicken skin collagen peptides and zinc sulfate *in vivo*

Zinc was reported to have concentration-dependent anti-tumor activity and to be proposed as an anti-tumor agent in treating cancers (8, 45). The above experiments showed that CCP–Zn has better bioavailability than ZnSO_4_, so we speculated whether CCP–Zn has better anti-tumor activity than inorganic zinc.

**FIGURE 5:**
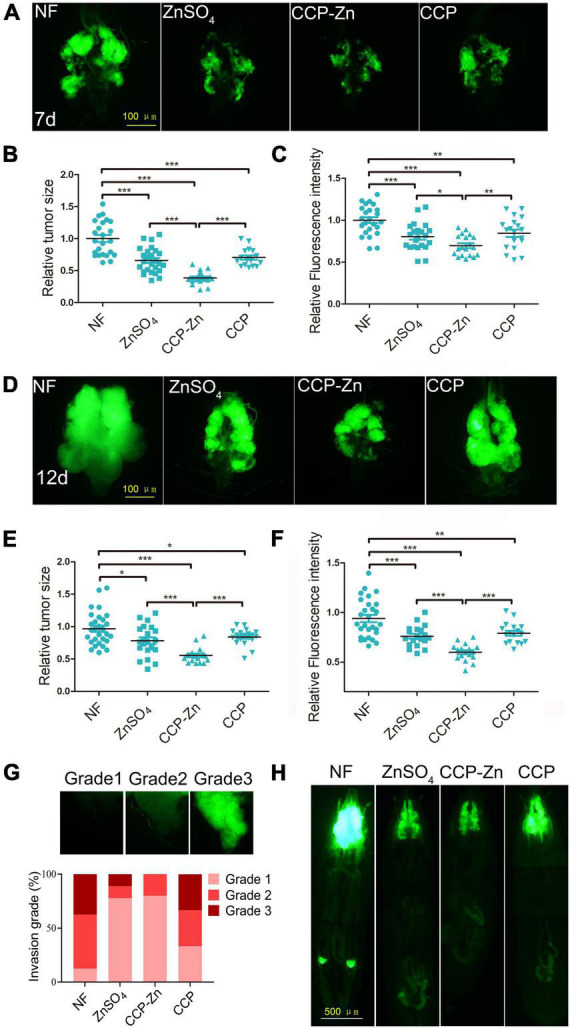
The anti-tumor activity of CCP-Zn is higher than CCP or ZnSO_4_. **(A)** Compared with tumor control, ZnSO_4_, CCP, or CCP–Zn alleviated tumor growth in the cephalic complex at day 7 after egg laying (AEL). **(B,C)** Quantification of tumor size and fluorescence intensity in the cephalic complex at day 7 AEL. *n* = 15. **(D)** Compared with tumor control, ZnSO_4_, CCP, or CCP–Zn alleviated tumor growth and invasion in the cephalic complex at day 12 AEL. **(E,F)** Quantification of tumor size and fluorescence intensity in the cephalic complex at day 12 AEL. *n* = 15. **(G)** Quantification of the degree of tumor invasion at day 12 AEL. *n* = 15. **(H)** Compared with tumor control, ZnSO_4_, CCP, or CCP–Zn alleviated tumor growth and invasion in the whole body at day 12 AEL. Data are represented as mean ± SEM of the biological replicates. *p < 0.05, **p < 0.01, ***p < 0.001; two-tailed Student’s *t*-test.

A classic tumor model driven by a combination of oncogenic Raf^GOF^ and scrib mutants in eye-antennal discs of *Drosophila* (28) was used to test the effect of ZnSO_4_, CCP, and CCP–Zn on the tumorigenesis and cancer progression of a malignant tumor. Previous reports showed that tumors in this model metastasize at around day 7 AEL and progressively invade other tissues until larval death at approximately day 15 (46). The mouth hooks and the ventral nerve cord (VNC) are two tissues that tumor cells initially migrate toward because these tissues are physically attached to the eye-antennal disc, the host tissue of the primary tumors (47). As shown in Figure 5A, GFP–labeled tumor clones are generated in the eye imaginal discs and the neuroepithelium of the brain in the head at 7 days AEL. ZnSO_4_, CCP–Zn, or CCP supplementation dramatically improved the hyper-proliferated tumors in the eye-antennal imaginal discs at 7 or 12 days AEL. Subsequently, the tumor size and fluorescence intensity in the cephalic complex of tumor larvae at 7 days AEL stage were measured. The results showed that ZnSO_4_, CCP–Zn, or CCP exhibited a strong reduction in the tumor size of the cephalic complex (with a 32, 61, and 29% decrease, respectively; Figure 5B) and the fluorescence intensity of cephalic complex (with 18, 27, and 12 % decrease, respectively; Figure 5C). At 12 days AEL stage, both the tumor growth and invasion were inhibited by ZnSO_4_, CCP–Zn, or CCP (Figure 5D). The tumor size of the cephalic complex was reduced by ZnSO_4_, CCP–Zn, or CCP (with a 21, 44, and 12% decrease, respectively; Figure 5E), and the fluorescence intensity of the cephalic complex was reduced by ZnSO_4_, CCP–Zn, or CCP (with 16, 33, and 13% decrease, respectively; Figure 5F). Besides, we found that almost every VNC was attacked by tumor cells in both tumor control and CCP group with varying degrees at 12 days AEL (Figures 5D,G), but fewer tumor cells metastasized to the VNC in the ZnSO_4_ group. Notably, almost no tumor clone exhibited an aggressive phenotype and penetrated VNC in CCP–Zn group. To sum up, these results suggest that CCP–Zn has better inhibitory activity on tumor growth and invasion than ZnSO_4_ or CCP *in vivo*.

Tumor metastasis is the major cause of cancer mortality (48). *Drosophila* may provide important evidence for clinical cancer treatment because the tumor metastasis could be detected in the whole body (49, 50). Tumor cells proliferated in the eye-antennal discs of the malignant Raf^GOF^scrib^–/–^ flies and migrated to several places, such as intestines, fat body, hemolymph, etc. (36, 51). To assess the effect of ZnSO_4_, CCP–Zn, or CCP on tumor metastasis, we observed the whole body of tumor flies at 12 days AEL using a fluorescence microscope (Figure 5H). The malignant phenotypes were restrained by ZnSO_4_, CCP, or CCP–Zn treatments (Figure 5H). Notably, CCP–Zn exhibited the strongest inhibitory effect on tumor growth, invasion, and migration (Figure 5H). Altogether, these data demonstrated that all three reagents could function as tumor suppressors, and the anti-tumor activity of CCP–Zn is better than ZnSO_4_ or CCP.

### Chicken skin collagen peptides–Zn restrains tumor progression by reducing the autonomous and non-autonomous autophagy levels

**FIGURE 6:**
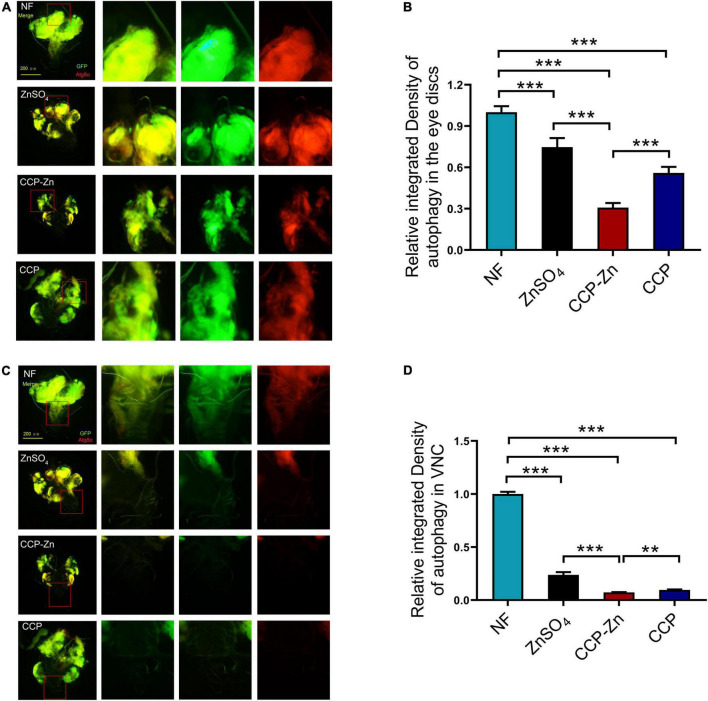
ZnSO_4_, CCP, or CCP–Zn inhibit tumor growth, invasion, and migration by suppressing autophagy in *Drosophila*. **(A)** ZnSO_4_, CCP, or CCP–Zn inhibits autonomous and non-autonomous autophagy in the eye discs of tumor *Drosophila*. **(B)** Quantification of tumor size and fluorescence intensity of **(A)**. **(C)** Activation of autophagy in VNC of tumor *Drosophila* at day 12 AEL can be rescued when they reared on ZnSO_4_, CCP, or CCP–Zn media. **(D)** Quantification of fluorescence intensity of **(C)**. Data are represented as mean ± SEM of the biological replicates. **p < 0.01, ***p < 0.001; two-tailed Student’s *t*-test.

Previous studies demonstrated that autophagy promotes tumor growth and invasion in human cancers (51, 52). The eye discs of Raf^GOF^scrib^–/–^ were reported to show robust autophagy in comparison to wild-type animals (51). Then, we investigated the effect of CCP–Zn on the autophagy level in the imaginal eye discs of these tumor clones (Figure 6). The results showed that both the autonomous (yellow signals in the merged images) and non-autonomous autophagy (red signals in the merged images) in Raf^GOF^scrib^–/–^ flies could be significantly restored by ZnSO_4_, CCP, or CCP–Zn (Figures 6A,B). As mentioned before, tumor cells initially migrate to VNC at 12 days AEL in the Raf^GOF^scrib^–/–^ model. When tumor cells metastasize to VNC, autophagy is accompanied by the formation of tumor cells (Figures 6C,D). The autophagy level, the tumor growth, as well as the invasion are rescued by ZnSO_4_, CCP, or CCP–Zn. CCP–Zn showed the best rescue effect. These data suggested that ZnSO_4_, CCP, or CCP-Zn restore the tumor overgrowth, invasion, and distant migration by inhibiting autonomous and non-autonomous autophagy.

## Discussion

In this study, we extracted CCP by step-by-step hydrolysis with bromelain and flavourzyme. The CCP displayed low molecular weights (<5 kDa), antioxidative activity, and high zinc-chelating ability. More importantly, we prepared novel CCP–Zn by chelating the CCP with ZnSO_4_. With the help of the *Drosophila* model, we found that the CCP–Zn has higher bioavailability and stronger anti-tumor activity than ZnSO_4_ *in vivo.* The mechanism studies indicated that both ZnSO_4_ and CCP–Zn could restore tumor growth and invasion by inhibiting the autonomous and non-autonomous autophagy in tumor cells and microenvironment.

Zinc deficiency is related to many disorders, such as anorexia, loss of appetite, smell and taste disorders (6). It is known that organic chelate zinc has advantages of low immunogenicity, good tissue permeability, high safety, and difficulty in accumulating in tissues. Chelation-based compounds containing polypeptide or an amino acid and zinc have attracted more and more attention because of their high bioavailability characteristics. By using a *Drosophila* model of zinc deficiency, we found that CCP–Zn showed better bioavailability than ZnSO_4_. Therefore, the CCP–Zn we prepared in this study may be used as a potential effective zinc supplement in the future.

Recently, most research studies on peptides with an anti-tumor activity focus on peptides composed of more than 50 amino acids, which are naturally occurring or artificially synthesized low molecular weight ligands (53, 54). Low molecular weight peptides exhibited many functions in cancer nanomedicine, including serving as drug carriers, targeting ligands, and protease-responsive substrates for drug delivery (54). Compared with antibodies or proteins, low molecular weight peptides have higher cell or tissue permeability, peptides can significantly improve the detection and cure rate of tumors (53, 55). Several peptide-based imaging probes and therapeutic agents have been used in clinical trials (56). Therefore, peptides have attracted more and more attention in tumor diagnosis and treatment because of their small size, high affinity, good stability, easy modification, and low immunogenicity (54). In this study, we found that the < 5 kDa CCP showed good anti-tumor activity. A lot of anti-tumor peptides have been found in recent years because of their advantages of targeting, specificity, safety, and so on (57, 58). The anti-tumor mechanisms of these peptides are diversified: direct anti-tumor effect can be achieved by inhibiting the proliferation of tumor cells and promoting the apoptosis of tumor cells, or indirect anti-tumor effect can be achieved by enhancing and stimulating the immune response of the body to tumor cells, or by inhibiting the formation of tumor blood vessels (58, 59). Besides, many studies have demonstrated that the anti-tumor effect of zinc could be potentially due to its differential regulation of host immune cells and cancer cells within the tumor microenvironment (60). Zinc deficiency results in inflammatory cytokines production, disrupts DNA–protein interaction, oxidative stress, apoptosis, and depresses the function of the immune response (61, 62). However, the role of zinc in conditioning the tumor microenvironment has not been investigated. Recent studies showed that autophagy in the tumor microenvironment promotes tumor growth by supplying nutrients and other factors, so the autophagy pathway is required for tumor progression (52). In this study, we found that CCP, ZnSO_4_, and CCP–Zn inhibit tumor growth and invasion by reducing the autonomous and non-autonomous autophagy activities; we speculated that CCP–Zn can inhibit tumor growth from two opposite sides: on the one hand, CCP–Zn inhibits the autonomous autophagy of tumor cells and reduces the sustainable energy of tumor growth. On the other hand, CCP–Zn inhibits the involuntary autophagy of surrounding normal cells and tumor growth. However, how CCP, ZnSO_4_, and CCP–Zn accomplish this task requires more mechanistic investigation. Numerous studies have shown that oxidative stress induced by reactive oxygen species (ROS) plays an essential regulatory role in tumor progression (63–65). Zinc deficiency induces oxidative stress; zinc supplementation reverses this effect (60). The CCP also exhibited antioxidant activity. Whether ROS is involved in the inhibition of CCP, ZnSO_4_, and CCP–Zn on autophagy and tumor progression needs further investigation. As shown in Figure 1, the DPPH radical scavenging activity of the sample is very low. Thus, we doubt that the anti-tumor activity of the CCP relies on its antioxidant properties. The underlying mechanisms need further investigation. Moreover, CCP–Zn exhibited stronger anti-tumor activity than CCP and ZnSO_4_. The zinc supplement therapeutic method has been proven to be good tumor adjuvant therapy. Therefore, the high bioavailability of CCP–Zn may be critical for its good anti-tumor activity. Taken together, these results raise the possibility that CCP–Zn, or more broadly organic chelate zinc, could be a new kind of promising therapeutic target in cancer pathology.

## Conclusion

In this study, low molecular weights collagen peptides (< 5 kDa) were isolated from chicken skin by step-by-step hydrolysis. Gly, Pro, Hyp, and Ala are the main amino acids in the CCP; the acidic amino acids and basic amino acids participated in the formation of the CCP–Zn chelate. We prepared novel CCP–Zn by chelating the CCP with ZnSO_4_. CCP and CCP–Zn showed antioxidant and anti-tumor activities. Moreover, CCP–Zn showed the highest bioavailability, antioxidant activity, and anti-tumor activity among CCP, ZnSO_4_, and CCP–Zn. Therefore, this research provides free technical support for higher-valued utilizing chicken by-products. CCP, ZnSO_4_, or CCP–Zn effectively inhibited the tumor growth, invasion, and migration in *Drosophila* malignant tumor model. In addition, CCP, ZnSO_4_, or CCP–Zn suppressed the autonomous and non-autonomous autophagy in tumor cells and microenvironment. Taken together, these findings provide *in vivo* evidence that CCP and CCP–Zn are expected to prevent cancer.

## Data availability statement

The original contributions presented in this study are included in the article/supplementary material, further inquiries can be directed to the corresponding author.

## Author contributions

TL and LZ: data curation. TL, LZ, XJ, and GX: formal analysis. GX: funding acquisition, project administration, supervision, writing—review and editing, and conceptualization. TL, XJ, and LZ: investigation, validation, writing—original draft, and methodology. All authors have read and agreed to the published version of the manuscript.
